# Dramatic and sustained regression of an inferior vena cava tumor thrombus in a patient with unresectable giant hepatocellular carcinoma following sequential systemic therapy: a case report and literature review

**DOI:** 10.3389/fonc.2026.1930045

**Published:** 2026-10-27

**Authors:** Ju Hou, Kun Li Wu

**Affiliations:** Department of Hepatobiliary Surgery, Guizhou Provincial People’s Hospital, Guiyang, Guizhou, China

**Keywords:** bevacizumab biosimilar, case report, hepatocellular carcinoma, immune checkpoint inhibitor, inferior vena cava tumor thrombus, regorafenib

## Abstract

Hepatocellular carcinoma (HCC) with major vascular invasion, particularly inferior vena cava (IVC) tumor thrombus, carries a dismal prognosis and has long been considered a treatment challenge. Although immune checkpoint inhibitors (ICIs) combined with anti-angiogenic agents have become the first-line standard systemic therapy for advanced HCC, data on long-term survival in patients with large tumors complicated by IVC tumor thrombus remain scarce. Herein, we report the case of a 63-year-old male patient with Barcelona Clinic Liver Cancer (BCLC) stage B unresectable HCC in whom an IVC tumor thrombus emerged during first-line therapy, with a baseline Child-Pugh score of 7 (class B) and albumin-bilirubin (ALBI) grade 3. The patient received first-line sintilimab plus the bevacizumab biosimilar IBI305 at an individualized 4-week interval for financial reasons. Early in first-line therapy (January 2023) a new IVC filling defect appeared; multidisciplinary review favored improved visualization of a previously occult thrombus over true progression, and treatment was continued. After twelve cycles, therapy was self-discontinued and the patient remained off treatment for 10.5 months, during which he required admission for massive ascites (at month 3) and the tumor later progressed. Regorafenib was then initiated as a non-standard second-line agent at an individualized reduced dose (80 mg/day, later 80 mg every other day for hand-foot skin reaction). The IVC tumor thrombus regressed completely and the target-lesion sum of diameters decreased by 48.9% from baseline, achieving partial response (PR) per RECIST 1.1 by June 2026. As of June 2026, the patient remains alive more than 4 years from initial detection of the liver mass. This case demonstrates that durable long-term survival can be achieved in patients with giant HCC and IVC tumor thrombus through sequential systemic therapy, even in the setting of treatment interruption. This report provides clinical insight for the management of advanced HCC with major vascular invasion.

## 1 Introduction

Hepatocellular carcinoma (HCC) is the sixth most common malignancy worldwide and the fourth leading cause of cancer-related death, with the disease burden particularly pronounced in East Asia due to chronic hepatitis B virus (HBV) infection (1, 2). Approximately 70%–80% of patients are diagnosed at an advanced stage, precluding curative resection or local ablation (3, 4). Among these, major vascular invasion—including portal vein tumor thrombus (PVTT) and inferior vena cava (IVC) tumor thrombus—occurs in 10%–40% of cases; without systemic treatment, median overall survival (OS) is only 2.7–4.0 months (5, 6). The presence of IVC tumor thrombus poses a particularly severe therapeutic challenge, as it may precipitate Budd-Chiari syndrome, pulmonary embolism, and rapid hemodynamic deterioration (2, 5).

Over the past decade, the emergence of molecularly targeted therapies and immune checkpoint inhibitors (ICIs) has fundamentally reshaped the treatment landscape for advanced HCC (4, 7, 8). The IMbrave150 trial established atezolizumab plus bevacizumab as the first-line standard of care, demonstrating superior OS and progression-free survival (PFS) compared with sorafenib (9, 10). In China, the combination of sintilimab (a PD-1 inhibitor) with the bevacizumab biosimilar IBI305 has shown comparable efficacy in the phase 2–3 ORIENT-32 trial and is widely used in clinical practice (11–13). Despite these advances, patients with giant HCC and IVC tumor thrombus continue to fare poorly, with objective response rates (ORR) typically below 30% and median OS rarely exceeding 12–18 months (5, 14).

Regorafenib, a multi-kinase inhibitor targeting VEGFR, TIE2, KIT, RET, RAF-1, BRAF, and FGFR, is approved for second-line treatment after sorafenib failure or intolerance (12, 15). While the RESORCE trial confirmed an OS benefit with regorafenib, its efficacy after ICI/anti-VEGF therapy, and in reversing large-vessel tumor thrombus, has not been well characterized (16, 17). Emerging evidence indicates that tyrosine kinase inhibitors (TKIs) can modulate the tumor immune microenvironment and may potentiate the efficacy of ICIs when used sequentially (18–20). Nevertheless, real-world data on long-term outcomes following sequential immune-targeted therapy in patients with giant HCC and IVC tumor thrombus remain limited (21, 22).

This article reports a 63-year-old male with HBV-related giant HCC and IVC tumor thrombus who achieved durable survival exceeding 4 years through intermittent systemic therapy, including sintilimab plus IBI305 followed by regorafenib. This case offers clinical insight into the sequential management of advanced HCC with major vascular invasion.

## 2 Case report

### 2.1 Patient presentation

A 63-year-old male was admitted on 22 October 2022 with a 10-month history of an incidentally discovered liver mass. He reported mild abdominal distension without weight loss. Past medical history included cholecystectomy 40 years prior. There was no history of hypertension, diabetes, coronary artery disease, psychiatric illness, or familial genetic disorders. No prior antitumor therapy had been received. Physical examination revealed a well-healed surgical scar on the abdomen; otherwise, no abnormalities were noted.

Admission laboratory tests revealed the following: alanine aminotransferase (ALT) 67 U/L (normal range 0–50 U/L); total bilirubin (T-BIL) 23.8 μmol/L (normal range 0–23 μmol/L); albumin 24.6 g/L (normal range 35–55 g/L); prothrombin time (PT) 13.9 s (prolongation 0 s). There was no ascites and no hepatic encephalopathy. Complete blood count was unremarkable. Hepatitis B surface antigen (HBsAg) was >250 IU/ml (chemiluminescence immunoassay; normal range 0–0.05 IU/ml), HBeAg was 0.44 S/CO (negative), and anti-HBe was 0.02 S/CO. The baseline Child-Pugh score was 7 (class B: bilirubin 1 point, albumin 3 points, PT prolongation 0 s 1 point, no ascites 1 point, no encephalopathy 1 point), corresponding to albumin-bilirubin (ALBI) grade 3. Pretreatment tumor markers, measured at an outside hospital on 27 September 2022, showed alpha-fetoprotein (AFP) 23.4 ng/ml (normal range 0–10 ng/ml) and protein induced by vitamin K absence or antagonist-II (PIVKA-II) 77.8 ng/ml (normal range 0–40 ng/ml).

Contrast-enhanced CT of the abdomen showed a bulky liver with multiple intrahepatic lesions. The two target lesions measured 13.51 cm and 6.67 cm in longest diameter (sum of diameters 20.18 cm), consistent with HCC imaging characteristics, accompanied by intrahepatic metastases (**Figure 1A**). Because the patient did not commence therapy until 22 October 2022 for personal reasons and no formal target-lesion measurement was made at admission, this earliest available complete scan was adopted as the RECIST 1.1 baseline. Based on the clinical history, elevated tumor markers, and imaging findings, intrahepatic cholangiocarcinoma and benign liver tumors were excluded. The pre-treatment diagnosis was unresectable HCC with multiple intrahepatic metastases, BCLC stage B (multinodular disease without macrovascular invasion or extrahepatic spread; deemed unsuitable for resection or TACE by multidisciplinary assessment and therefore treated by stage-migrated systemic therapy), Child-Pugh class B, and Eastern Cooperative Oncology Group (ECOG) performance status 0. No definite tumor thrombus was identified in the IVC before treatment (**Figures 1A**, **2A**); the IVC filling defect was first detected on 15 January 2023, early in first-line therapy (Section 2.2).

**Figure 1:**
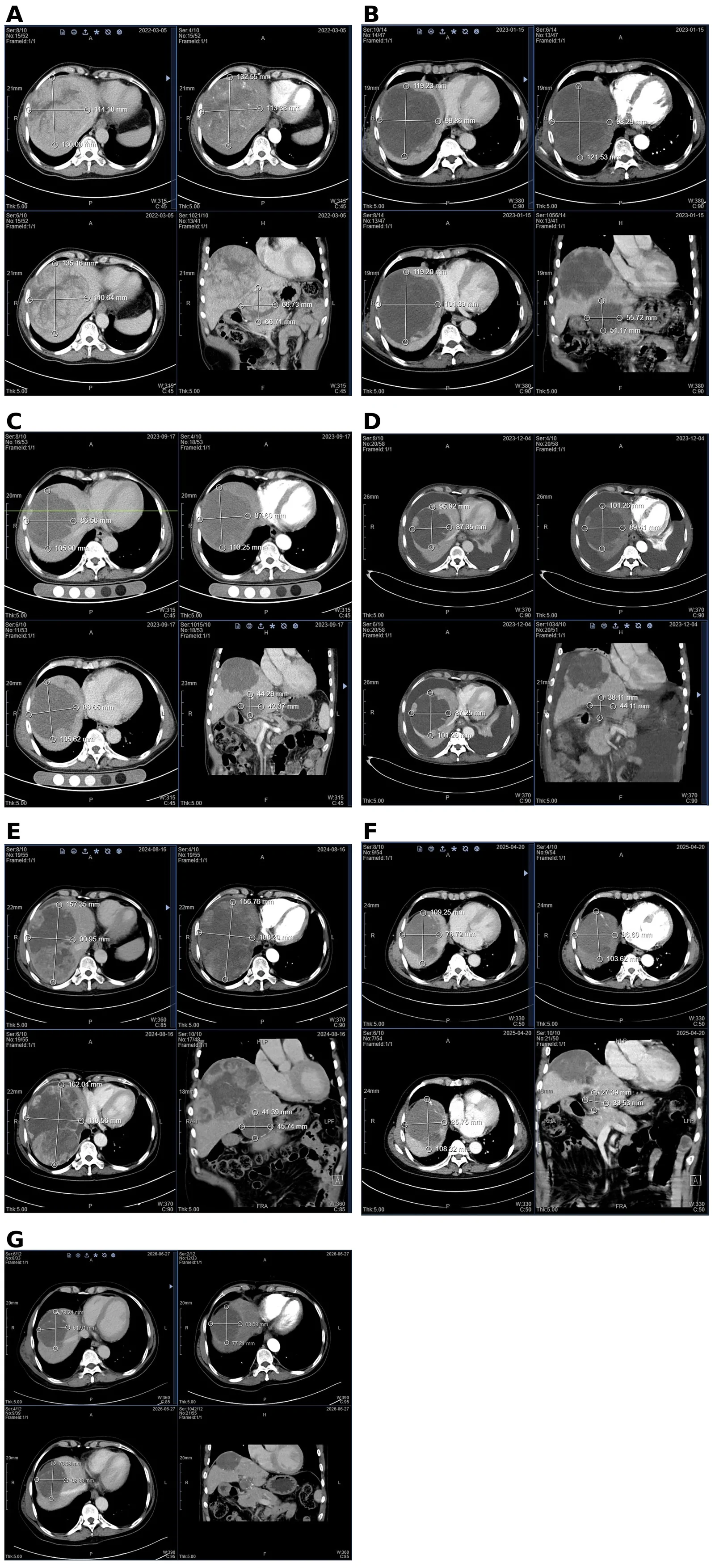
Contrast-enhanced CT images of the dominant hepatic lesions at different time points. **(A)** Baseline scan (5 March 2022) showing multiple hepatic lesions; the two target lesions measured 13.51 cm and 6.67 cm (sum of diameters 20.18 cm) with marked arterial hyperenhancement and relative washout on the venous phase. **(B)** 15 January 2023, after approximately 3 months of sintilimab plus IBI305: target lesions 12.15 cm and 5.57 cm (sum 17.72 cm, −12.2% from baseline); a new IVC filling defect was noted. **(C)** 17 September 2023, after twelve cycles: target lesions 11.02 cm and 4.42 cm (sum 15.44 cm, −23.5% from baseline), with a stable IVC thrombus, although the overall first-line assessment remained PD because of the new lesion detected on 15 January 2023. **(D)** 4 December 2023, approximately 3 months into the treatment interruption: tumor burden similar to the prior scan but massive ascites had appeared; the IVC thrombus was unchanged. **(E)** 16 August 2024, after the 10.5-month interruption and 1 month after rechallenge: the target lesions progressed to 16.20 cm and 4.57 cm (sum 20.77 cm, a 34.5% increase from the nadir of 15.44 cm), meeting PD by RECIST 1.1; the IVC thrombus was faintly visualized, prompting the switch to regorafenib. **(F)** 20 April 2025, on regorafenib: target lesions 10.92 cm and 3.35 cm (sum 14.27 cm, −29.3% from baseline), with complete regression of the IVC thrombus. **(G)** 27 June 2026: continued regression to 7.82 cm and 2.50 cm (sum 10.32 cm, −48.9% from baseline), formally achieving partial response by RECIST 1.1; the IVC thrombus had disappeared.

**Figure 2:**
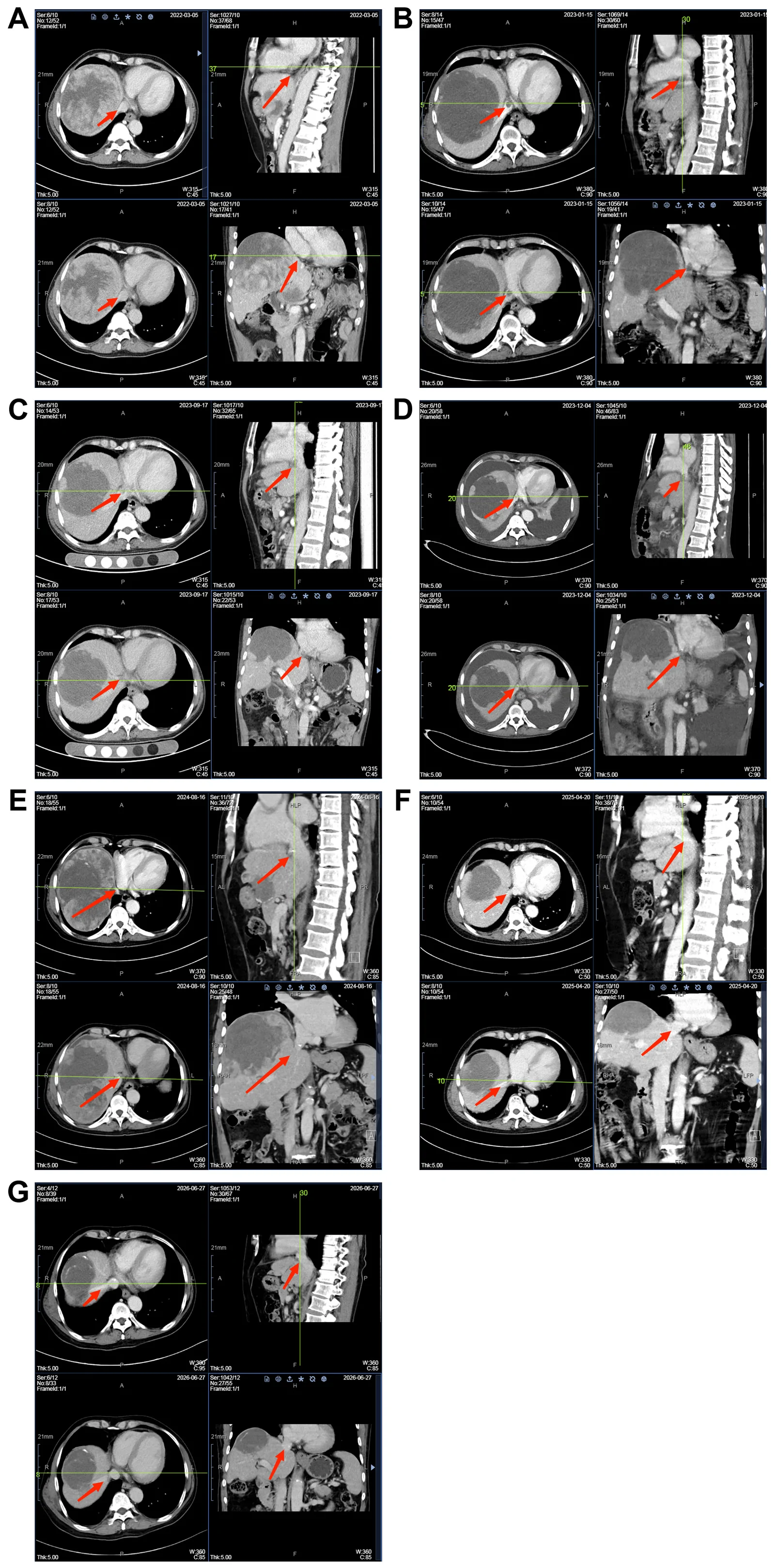
Serial contrast-enhanced CT (venous phase, axial and sagittal/coronal reconstructions) showing the IVC tumor thrombus (red arrows) at the same time points as **Figure 1**. **(A)** 5 March 2022, pretreatment: no IVC thrombus. **(B)** 15 January 2023: new intraluminal IVC filling defect (arrow), consistent with tumor thrombus. **(C)** 17 September 2023: thrombus stable. **(D)** 4 December 2023: thrombus stable. **(E)** 16 August 2024: thrombus faintly visualized. **(F)** 20 April 2025: thrombus completely regressed. **(G)** 27 June 2026: no visible thrombus. Tumor thrombus was distinguished from bland thrombus by its arterial-phase enhancement, expansion of the IVC lumen, and continuity with the intrahepatic tumor, whereas bland thrombus typically shows no enhancement. Because the enhancing thrombus blended with the opacified blood pool on venous-phase images, no reproducible caliper measurement could be defined; thrombus response was therefore assessed qualitatively (appearance, stability, and regression), and no baseline thrombus measurement was recorded.

Multidisciplinary assessment identified two tumor foci involving liver segments 2, 4, 5, 7, and 8, with suspected invasion of segments 3 and 6. Surgical resection was not feasible. The patient and his family declined surgery and interventional therapy, and requested systemic targeted therapy combined with immunotherapy. Because of financial constraints, the patient also declined long-term oral antiviral therapy for HBV despite counseling; no baseline HBV DNA was available, and no serial HBV DNA monitoring was performed thereafter. After comprehensive discussion of potential complications, adverse effects, and the possibility of tumor progression despite treatment, informed consent was obtained.

### 2.2 First-line therapy: sintilimab plus IBI305

Following multidisciplinary team discussion and informed consent, the patient commenced first-line systemic therapy on 22 October 2022 with sintilimab (200 mg, intravenous infusion) plus the bevacizumab biosimilar IBI305 (15 mg/kg, intravenous infusion), administered every 4 weeks. Because of financial constraints, a 4-week interval was used instead of the standard 3-week schedule of the ORIENT-32 regimen (13). The initial treatment cycles were well tolerated, with no significant adverse reactions observed.

Follow-up CT on 15 January 2023, after approximately 3 months of therapy, showed the target-lesion sum of diameters had decreased from 20.18 cm to 17.72 cm (12.15 cm + 5.57 cm), a reduction of 12.2% from baseline (**Figure 1B**). Serum AFP had declined to 13.4 ng/ml and PIVKA-II to 67.82 ng/ml; albumin had risen to 37.8 g/L and bilirubin had normalized to 14.2 μmol/L, with recovery of liver function to Child-Pugh class A (5 points). Notably, a new filling defect in the IVC was detected on this scan (**Figure 2B**). Because the appearance of a new lesion meets the definition of progressive disease (PD) under RECIST 1.1, this finding was formally recorded as PD; however, multidisciplinary review considered it more likely to represent improved visualization of a previously occult thrombus than true progression, given the concurrent shrinkage of intrahepatic lesions, the decline in tumor markers, and the patient’s preserved performance status. Treatment was therefore continued. ECOG performance status remained 0.

The patient continued treatment, with administrations given every 4 weeks on schedule, without delays or missed doses; the 12th and final administration was on 26 August 2023. Follow-up CT on 17 September 2023, after 12 cycles, showed a further decrease of the target-lesion sum of diameters to 15.44 cm (11.02 cm + 4.42 cm), a 23.5% reduction from baseline; the IVC tumor thrombus remained stable, and no new lesions appeared; the target lesions had shrunk by 23.5% from baseline, although the overall first-line assessment remained PD under RECIST 1.1 because of the new lesion first detected on 15 January 2023 (**Figure 1C**). Serum AFP was 10.3 ng/ml and PIVKA-II was 602.37 ng/ml; albumin was 34.4 g/L and bilirubin 12.8 μmol/L (Child-Pugh class A, 6 points). ECOG performance status remained 0. During bevacizumab-containing therapy the patient developed elevated blood pressure, which was neither monitored regularly nor treated with regular antihypertensive medication.

### 2.3 Treatment interruption and disease progression

After the twelfth cycle (last administration 26 August 2023), the patient self-discontinued systemic therapy because of financial constraints, while concurrently taking oral traditional Chinese medicine. Approximately 3 months into this interruption, in December 2023, he was re-admitted with progressive abdominal distension and bilateral lower-extremity edema.

Physical examination revealed shifting dullness and massive ascites. Laboratory tests showed albumin 28.5 g/L and bilirubin 8.6 μmol/L. Abdominal ultrasound confirmed bilateral pleural effusions and massive ascites. CT on 4 December 2023 showed tumor burden similar to the 17 September 2023 assessment, with new-onset peritoneal fluid and extensive ascites (**Figure 1D**); the IVC tumor thrombus showed no change. Liver function had deteriorated to Child-Pugh class B, and ECOG performance status declined to 2.

The patient underwent therapeutic paracentesis, albumin supplementation, and diuretic therapy. After symptomatic improvement, he was discharged with close follow-up. On 15 May 2024, CT showed ascites reduced from the December 2023 assessment; albumin had recovered to 34.9 g/L and bilirubin to 12.5 μmol/L, with recovery of liver function to Child-Pugh class A and improvement of ECOG performance status to 0.

### 2.4 Rechallenge and switch to second-line therapy

By 15 July 2024, the patient had been off immunotherapy and targeted therapy for 10.5 months in total (from 26 August 2023, the last administration). Laboratory tests at that time showed AFP 58.9 ng/ml (having peaked at 73.8 ng/ml in June 2024) and PIVKA-II 16,934.56 ng/ml, indicating marked tumor marker elevation. After thorough discussion with the patient and his family, sintilimab plus IBI305 was re-initiated.

However, CT on 16 August 2024 revealed tumor progression, with the target-lesion sum of diameters increasing to 20.77 cm (16.20 cm + 4.57 cm), a 34.5% increase from the lowest value of 15.44 cm, meeting the definition of PD by RECIST 1.1 (**Figure 1E**). The IVC tumor thrombus had become faintly visualized. The treatment regimen was therefore switched to regorafenib. Because of financial constraints, an individualized starting dose of 80 mg orally once daily was chosen rather than the standard 160 mg/day. Because of grade 2 hand-foot skin reaction (HFSR), the dose was adjusted to 80 mg every other day, with symptomatic supportive care. The patient demonstrated good adherence to the adjusted regimen.

### 2.5 Response to regorafenib and current status

CT on 20 April 2025 showed marked tumor regression, with the target-lesion sum of diameters decreasing to 14.27 cm (10.92 cm + 3.35 cm), a 29.3% reduction from baseline and just short of the 30% threshold for partial response (**Figure 1F**). Most notably, the IVC tumor thrombus had completely regressed. Serum AFP had normalized to 5.0 ng/ml and PIVKA-II had decreased to 249.23 ng/ml. Liver function remained Child-Pugh class A, and ECOG performance status was 0.

The most recent CT on 16 January 2026 showed continued tumor shrinkage to a maximum diameter of 8.1 cm, with AFP 43.1 ng/ml and PIVKA-II 146.15 ng/ml. The latest CT on 27 June 2026 showed continued regression, with the target-lesion sum of diameters further decreasing to 10.32 cm (7.82 cm + 2.50 cm), a 48.9% reduction from baseline, formally achieving partial response (PR) by RECIST 1.1 (**Figure 1G**); the IVC tumor thrombus had disappeared. The patient continues regorafenib therapy at 80 mg every other day; the HFSR is manageable, and performance status remains good. As of June 2026, the patient remains alive more than 4 years from initial detection of the liver mass (**Figures 2A–G** and **Figures 3A–F** ).

**Figure 3:**
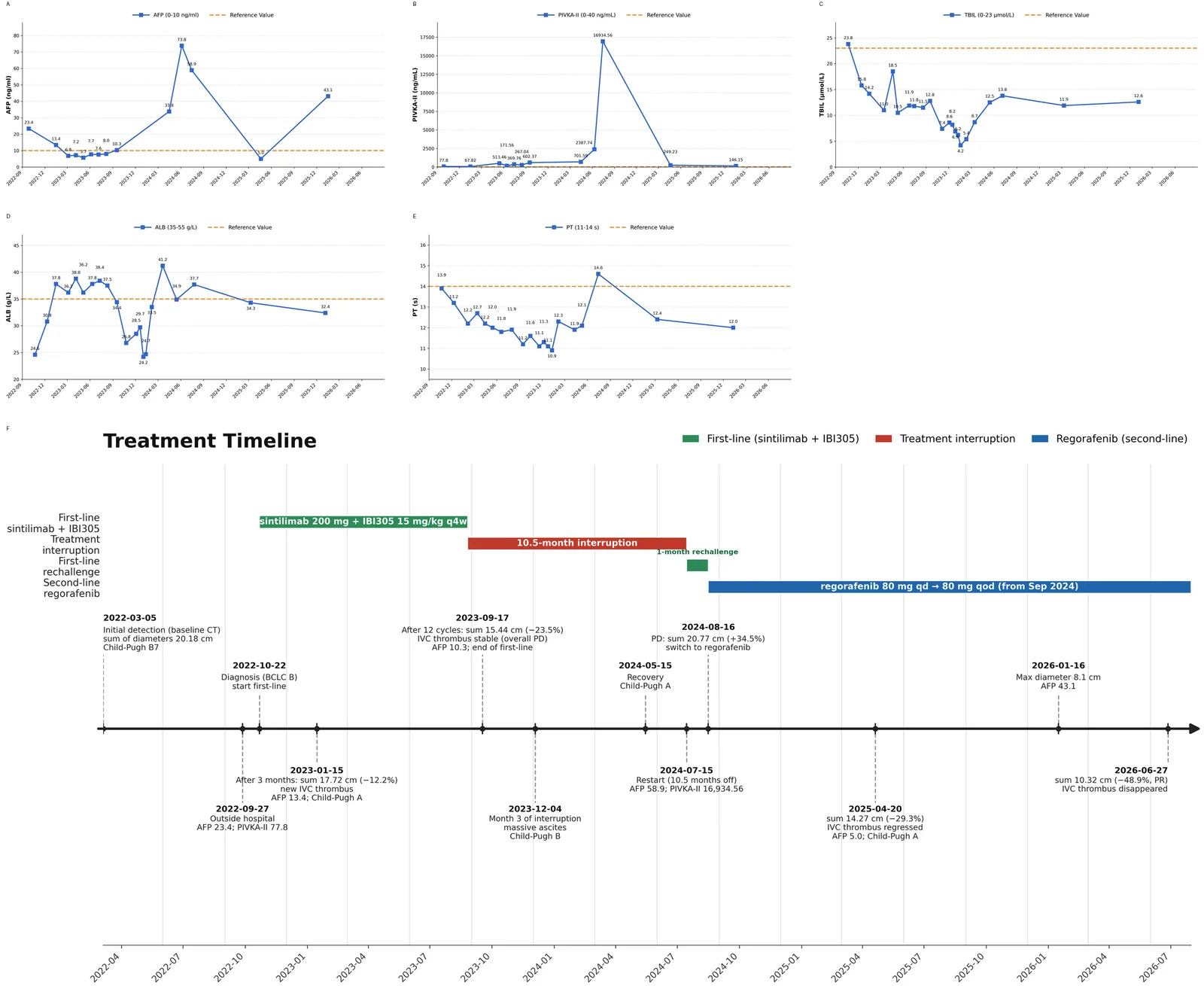
Serial changes in serum AFP **(A)**, PIVKA-II **(B)**, total bilirubin **(C)**, albumin **(D)**, and PT **(E)** throughout treatment, with baseline values from 27 September 2022; and the treatment timeline **(F)**. Dashed lines mark reference values. The timeline **(F)** indicates exact dates, treatment regimens and doses, RECIST 1.1 assessments, Child-Pugh class, AFP/PIVKA-II values, and the treatment interruption, so that the clinical course can be followed independently.

## 3 Discussion

This case describes a patient with HBV-related giant HCC and IVC tumor thrombus who achieved durable survival exceeding 4 years from initial lesion detection through sequential systemic therapy, despite a 10.5-month treatment interruption and progression on first-line immunotherapy. Several observations merit discussion.

First, the 23.5% reduction in target-lesion diameters achieved during twelve cycles of sintilimab plus IBI305 in the setting of a 13.5 cm tumor with IVC tumor thrombus is consistent with real-world evidence for ICI-based combination regimens in advanced HCC (7–9, 23). The ORBITA concept and subsequent analyses have demonstrated that anti-angiogenic agents combined with PD-1/PD-L1 inhibitors can promote vascular normalization and immune cell infiltration, thereby improving objective response rates (3, 24, 25). The decline in AFP and PIVKA-II in this patient further supports the biological activity of this combination (10, 14). We acknowledge that our patient’s interval was extended to 4 weeks rather than the 3-week schedule used in ORIENT-32 owing to financial constraints (13), Because the new IVC filling defect detected on 15 January 2023 constitutes a new lesion, the overall first-line assessment was PD under RECIST 1.1; although the target lesions continued to shrink with continued therapy, the formal criteria for stable disease or partial response were not met once progression had been recorded. It should also be noted that the IVC filling defect first detected on 15 January 2023 was formally classified as PD under RECIST 1.1 because new lesions define progression; the decision to continue therapy was based on multidisciplinary assessment that favored improved visualization of an occult thrombus over true progression, supported by concurrent marker decline and lesion shrinkage. This highlights the known difficulty of applying RECIST 1.1 to vascular tumor thrombi in HCC.

Second, the 10.5-month treatment interruption represented a critical turning point. The emergence of massive ascites at month 3 of the interruption, deterioration to Child-Pugh class B, and the dramatic rise in PIVKA-II to 16,934.56 ng/ml upon rechallenge underscore the aggressive biology of HCC with major vascular invasion and the importance of treatment continuity (2, 5). The progression to a sum of diameters of 20.77 cm upon re-administration of sintilimab plus IBI305 indicates the development of acquired resistance or accelerated disease evolution during the treatment-free interval. This observation aligns with the view that treatment interruption in advanced HCC can lead to rapid clinical deterioration, as the tumor microenvironment may shift toward an immunosuppressive phenotype during periods of absent therapeutic pressure (19, 20, 24).

Third, the switch to regorafenib produced not only tumor shrinkage but also complete regression of the IVC tumor thrombus. We emphasize that this sequence—regorafenib after PD-1/anti-VEGF therapy, at a reduced starting dose of 80 mg/day, later reduced to 80 mg every other day—was a non-standard, individualized strategy rather than an established second-line standard, which was historically defined after sorafenib failure (12, 15, 16). Encouragingly, recent prospective and real-world data support the activity of regorafenib after atezolizumab plus bevacizumab: a multicenter phase 2 trial reported median PFS of 3.5 months and OS of 10.5 months in this setting (26), and a real-world cohort confirmed that preserved liver function is a key determinant of the ability to receive second-line therapy at all (27). The complete regression of the IVC tumor thrombus in our patient is also not entirely without precedent: regorafenib-associated shrinkage of an HCC thrombus extending from the hepatic vein into the IVC, permitting subsequent conversion surgery, has been reported (28), and a systematic review of HCC with hepatic vein/IVC/right atrial thrombus documented that combined local and systemic treatment achieves the most favorable long-term survival (29). Our case extends this evidence by showing sustained, complete thrombus regression with systemic therapy alone over more than 2 years.

The mechanism underlying the response to regorafenib may be multifactorial, although it remains speculative in the absence of serial biopsy or immune profiling. The broad kinase inhibition profile of regorafenib—including VEGFR1-3, TIE2, KIT, RET, RAF-1, BRAF, PDGFR, and FGFR—may exert potent anti-angiogenic effects on the tumor thrombus vasculature, leading to thrombus devascularization and regression (6, 15, 18). TKIs have also been shown to modulate the tumor immune microenvironment by reducing regulatory T cells, promoting dendritic cell maturation, and inducing M1 macrophage polarization, potentially “priming” the tumor for enhanced immune surveillance (18–20); whether prior ICI exposure created such a window in this patient cannot be determined from our data. Beyond angiogenic and immune effects, recent mechanistic studies indicate that ferroptosis may contribute to regorafenib response and resistance: regorafenib can induce NOX4-dependent lipid peroxidation and ferroptosis (demonstrated in acute myeloid leukemia and hypothesized in HCC) (30), while HCC may evade regorafenib through ZNF207-driven PRDX1 lactylation and NRF2 activation (31) or through LARS1/GPX4 up-regulation and enhanced drug glucuronidation (32). We did not demonstrate ferroptosis in this patient, and we raise these pathways only as hypotheses consistent with the broader signaling landscape of HCC (33).

The sustained tumor shrinkage from a sum of diameters of 20.77 cm to 10.32 cm over 22 months of regorafenib therapy challenges the conventional notion that second-line TKIs can only delay progression without producing meaningful tumor regression (34). The depth and durability of the observed response—formal PR (−48.9%) by June 2026 with continued shrinkage—support the exploration of prolonged TKI therapy in responders, with careful toxicity management (12, 17, 35). The dose adjustment to 80 mg every other day because of HFSR highlights the importance of individualized toxicity management to preserve treatment continuity (35, 36).

The hepatic functional evolution observed in this patient is also noteworthy. The baseline Child-Pugh score of 7 reflected severe hypoalbuminemia (24.6 g/L); recovery to Child-Pugh class A during first-line therapy, transient deterioration to class B during the treatment interruption with onset of ascites, and subsequent recovery to class A after tumor recontrol indicate that hepatic decompensation in this setting may be reversible when tumor burden and portal-venous congestion are controlled (2, 36). This observation supports the view that systemic treatment efficacy and liver functional preservation are closely linked in advanced HCC.

From a broader perspective, this case contributes to the growing evidence that long-term survival is achievable in advanced HCC, even in traditionally poor-prognosis subgroups such as patients with large tumors and IVC tumor thrombus (5, 8, 14, 29). The observed survival of more than 4 years from initial lesion detection far exceeds the expected median OS for BCLC stage C HCC with vascular invasion (37) and is comparable to outcomes reported in selected cohorts of combination immunotherapy trials (1, 2, 9). However, it must be acknowledged that this is a single case, and the favorable outcome may have been influenced by patient-specific factors, including preserved performance status and restored treatment adherence.

Several limitations of this report should be noted. The absence of histopathological confirmation of HCC is a methodological limitation; although imaging characteristics and AFP/PIVKA-II elevation strongly support the diagnosis. The treatment interruption was self-imposed by the patient rather than protocol-driven, precluding formal analysis of its impact. Baseline endoscopic evaluation of varices, formal bleeding-risk assessment, and urinalysis for proteinuria were not performed before bevacizumab administration, and blood pressure was not monitored regularly; hypertension emerged during therapy and was not treated regularly—an important safety gap given the bleeding and proteinuric risks of anti-VEGF therapy (38). Because the patient was HBsAg-positive and declined antiviral therapy, HBV reactivation was a concrete risk during prolonged ICI therapy; however, baseline HBV DNA was not measured and no serial viral-load monitoring was performed, so reactivation cannot be excluded or confirmed. Current guidance recommends antiviral prophylaxis or regular HBV DNA monitoring in HBsAg-positive patients receiving immunosuppressive or immunotherapy-based treatment (39); the inability to implement this in our patient reflects real-world adherence barriers and should be regarded as a limitation rather than a model of care. The IVC tumor thrombus was assessed qualitatively rather than by serial caliper measurement, because no reproducible diameter could be defined against the opacified blood pool on venous-phase images. Finally, without serial tumor biopsies and molecular profiling, the mechanisms underlying the response to regorafenib remain speculative. Future studies incorporating multi-omics analysis and immune monitoring may help elucidate the biological basis of such exceptional responses (25, 40, 41).

## 4 Conclusion

This case reports a patient with giant HCC and IVC tumor thrombus who achieved durable survival exceeding 4 years from initial lesion detection through sequential sintilimab plus IBI305 followed by non-standard, individualized regorafenib therapy, despite a 10.5-month treatment interruption. The complete regression of the IVC tumor thrombus and sustained tumor shrinkage culminating in a formal partial response under second-line regorafenib highlight the possibility of meaningful long-term survival in this poor-prognosis population.

## Data Availability

The original contributions presented in the study are included in the article/supplementary material. Further inquiries can be directed to the corresponding author.

## References

[1] Yang X Yang C Zhang S Geng H Zhu AX Bernards R . Precision treatment in advanced hepatocellular carcinoma. Cancer Cell. (2024) 42:180–97. doi: 10.1016/j.ccell.2024.01.007 38350421

[2] Llovet JM Pinyol R Kelley RK El-Khoueiry A Reeves HL Wang XW . Molecular pathogenesis and systemic therapies for hepatocellular carcinoma. Nat Cancer. (2022) 3:386–401. doi: 10.1038/s43018-022-00357-2 35484418 PMC9060366

[3] Donne R Lujambio A . The liver cancer immune microenvironment: Therapeutic implications for hepatocellular carcinoma. Hepatology. (2023) 77:1773–96. doi: 10.1002/hep.32740 35989535 PMC9941399

[4] Llovet JM Montal R Sia D Finn RS . Molecular therapies and precision medicine for hepatocellular carcinoma. Nat Rev Clin Oncol. (2018) 15:599–616. doi: 10.1038/s41571-018-0073-4 30061739 PMC12452113

[5] Moris D Martinino A Schiltz S Allen PJ Barbas A Sudan D . Advances in the treatment of hepatocellular carcinoma: An overview of the current and evolving therapeutic landscape for clinicians. CA Cancer J Clin. (2025) 75:498–527. doi: 10.3322/caac.70018 40392748 PMC12593328

[6] Liu Z Lin Y Zhang J Zhang Y Li Y Liu Z . Molecular targeted and immune checkpoint therapy for advanced hepatocellular carcinoma. J Exp Clin Cancer Res. (2019) 38:447. doi: 10.1186/s13046-019-1412-8 31684985 PMC6827249

[7] Liu J Park K Shen Z Lee H Geetha P Pakyari M . Immunotherapy, targeted therapy, and their cross talks in hepatocellular carcinoma. Front Immunol. (2023) 14:1285370. doi: 10.3389/fimmu.2023.1285370 38173713 PMC10762788

[8] Hao L Li S Ye F Wang H Zhong Y Zhang X . The current status and future of targeted-immune combination for hepatocellular carcinoma. Front Immunol. (2024) 15:1418965. doi: 10.3389/fimmu.2024.1418965 39161764 PMC11330771

[9] Liu K Zhu Y Zhu H . Immunotherapy or targeted therapy as the first-line strategies for unresectable hepatocellular carcinoma: A network meta-analysis and cost-effectiveness analysis. Front Immunol. (2023) 13:1103055. doi: 10.3389/fimmu.2022.1103055 36713376 PMC9874298

[10] Li Q Li H Ma H Chen W . Efficacy and safety of first-line immunotherapy and targeted therapy in advanced HCC: A network meta-analysis with subgroup analysis based on HBV and HCV infection. Front Immunol. (2026) 17:1706446. doi: 10.3389/fimmu.2026.1706446 41694404 PMC12894325

[11] Fang H Ke Q Wu S Tu Q Wang L . Immune-targeted therapy with transarterial chemo(embolization) for unresectable HCC: A systematic review and meta-analysis. Front Immunol. (2024) 15:1421520. doi: 10.3389/fimmu.2024.1421520 39156893 PMC11329924

[12] Dong H Zhang Z Ni M Xu X Luo Y Wang Y . The trend of the treatment of advanced hepatocellular carcinoma: Combination of immunotherapy and targeted therapy. Curr Treat Options Oncol. (2024) 25:1239–56. doi: 10.1007/s11864-024-01246-9 39259476 PMC11485193

[13] Ren Z Xu J Bai Y Xu A Cang S Du C . Sintilimab plus a bevacizumab biosimilar (IBI305) versus sorafenib in unresectable hepatocellular carcinoma (ORIENT-32): A randomised, open-label, phase 2-3 study. Lancet Oncol. (2021) 22:977–90. doi: 10.1016/S1470-2045(21)00252-7 34143971

[14] Zhang C Xu Y Cai L Zhou S Feng J Fu S . Efficacy of combined TAE, HAIC, targeted, and immunotherapy in unresectable HCC: A multicenter propensity score matching study. Sci Rep. (2025) 15:21184. doi: 10.1038/s41598-025-08170-4 40595149 PMC12216019

[15] Wang T Zhang Q Wang N Liu Z Zhang B Zhao Y . Research progresses of targeted therapy and immunotherapy for hepatocellular carcinoma. Curr Med Chem. (2021) 28:3107–46. doi: 10.2174/0929867327666201013162144 33050856

[16] Yang F Deng K Zheng H Liu Z Zheng Y . Progress of targeted and immunotherapy for hepatocellular carcinoma and the application of next-generation sequencing. Ann Hepatol. (2022) 27:100677. doi: 10.1016/j.aohep.2022.100677 35093601

[17] Vogel A Saborowski A . Medical therapy of HCC. J Hepatol. (2022) 76:208–10. doi: 10.1016/j.jhep.2021.05.017 34538616

[18] Liu M Li L Cao L Li W Gu X Yang M . Targeted delivery of CCL3 reprograms macrophage antigen presentation and enhances the efficacy of immune checkpoint blockade therapy in hepatocellular carcinoma. J Immunother Cancer. (2025) 13:e010947. doi: 10.1136/jitc-2024-010947 39988347 PMC11848677

[19] Xie D Liu Y Xu F Dang Z Li M Zhang Q . Immune microenvironment and immunotherapy in hepatocellular carcinoma: mechanisms and advances. Front Immunol. (2025) 16:1581098. doi: 10.3389/fimmu.2025.1581098 40242773 PMC12000014

[20] Dai S Chen Y Cai W Dong S Zhao J Chen L . Combination immunotherapy in hepatocellular carcinoma: Synergies among immune checkpoints, TKIs, and chemotherapy. J Hematol Oncol. (2025) 18:85. doi: 10.1186/s13045-025-01739-6 41013723 PMC12465164

[21] Du JJ Yang F Dong W Zhang ZY Cheng Q Zhang EL . Revisiting the role of local treatments in the era of targeted therapy and immunotherapy for hepatocellular carcinoma. World J Surg Oncol. (2025) 23:323. doi: 10.1186/s12957-025-03963-3 40866921 PMC12382181

[22] Wan J Tang B Zhang W Zhou Y Jin C Yu C . Sequential HAIC-TACE combined with targeted and immunotherapy in hepatocellular carcinoma: A quality improvement study. Int J Surg. (2025) 111:6942–60. doi: 10.1097/JS9.0000000000002890 40693963 PMC12527709

[23] Yin Z Li X . Immunotherapy for hepatocellular carcinoma. Cancer Lett. (2020) 470:8–17. doi: 10.1016/j.canlet.2019.12.002 31811905

[24] Oura K Morishita A Tani J Masaki T . Tumor immune microenvironment and immunosuppressive therapy in hepatocellular carcinoma: A review. Int J Mol Sci. (2021) 22:5801. doi: 10.3390/ijms22115801 34071550 PMC8198390

[25] Li Y Li F Xu L Shi X Xue H Liu J . Single cell analyses reveal the PD-1 blockade response-related immune features in hepatocellular carcinoma. Theranostics. (2024) 14:3526–47. doi: 10.7150/thno.95971 38948071 PMC11209711

[26] Cheon J Ryoo BY Chon HJ Kim HD Ryu MH Kim KP . Multicenter phase 2 trial of second-line regorafenib in uHCC after progression on atezolizumab plus bevacizumab. Liver Cancer. (2025) 14:446–55. doi: 10.1159/000543666 40831890 PMC12360742

[27] Ben Khaled N Zarka V Hobeika B Schneider J Rau M Weich A . Therapeutic sequences of systemic therapy after Atezo+Bev for HCC: Real-world analysis of the IMMUreal cohort. Aliment Pharmacol Ther. (2025) 61:1755–66. doi: 10.1111/apt.70090 40181694 PMC12074566

[28] Takeda K Takeda M Kodama K Kaji R Yamashita T . Treatment of HCC with hepatic vein tumor thrombosis protruding into the IVC by conversion surgery following chemotherapy with regorafenib: A case report. Clin J Gastroenterol. (2020) 13:428–33. doi: 10.1007/s12328-019-01077-4 31970661 PMC7239798

[29] Wang QB Li J Zhang ZJ Li YK Liang YB Chen XM . The effectiveness and safety of therapies for HCC with tumor thrombus in the hepatic vein, IVC and/or right atrium: A systematic review and single-arm meta-analysis. Expert Rev Anticancer Ther. (2025) 25:561–70. doi: 10.1080/14737140.2025.2489651 40181594

[30] Mu R Pei X Xu N Liu B Liu L Li H . Regorafenib promotes ferroptosis in acute myeloid leukemia by upregulating NOX4. Biochem Biophys Res Commun. (2025) 792:152981. doi: 10.1016/j.bbrc.2025.152981 41242297

[31] Yang T Zhang S Nie K Cheng C Peng X Huo J . ZNF207-driven PRDX1 lactylation and NRF2 activation in regorafenib resistance and ferroptosis evasion. Drug Resist Update. (2025) 82:101274. doi: 10.1016/j.drup.2025.101274 40680452

[32] Tsai TY Lin HH Cheng YC Yadav VK Ko CC Cherng YG . LARS inhibition overcomes regorafenib resistance in HCC via ferroptosis and drug metabolism. Eur J Pharm Sci. (2026) 225:107614. doi: 10.1016/j.ejps.2026.107614 42476473

[33] Zheng J Wang S Xia L Sun Z Chan KM Bernards R . Hepatocellular carcinoma: Signaling pathways and therapeutic advances. Signal Transduct Target Ther. (2025) 10:35. doi: 10.1038/s41392-024-02075-w 39915447 PMC11802921

[34] Thomas MB O'Beirne JP Furuse J Chan AT Abou-Alfa G Johnson P . Systemic therapy for hepatocellular carcinoma: Cytotoxic chemotherapy, targeted therapy and immunotherapy. Ann Surg Oncol. (2008) 15:1008–14. doi: 10.1245/s10434-007-9705-0 18236117

[35] Feng J Zhao Y Zhai L Zhou J . Efficacy and safety of transarterial chemoembolization combined with targeted therapy and immunotherapy versus with targeted monotherapy in unresectable hepatocellular carcinoma: A systematic review and meta-analysis. Med (Baltimore). (2024) 103:e38037. doi: 10.1097/MD.0000000000038037 38701263 PMC11062670

[36] Nan YM Liu LD Zhao SX . Emphasis on targeted and immunotherapy for liver injury in hepatocellular carcinoma. Zhonghua Gan Zang Bing Za Zhi. (2023) 31:1121–4. doi: 10.3760/cma.j.cn501113-20230915-00108 38238942 PMC12813779

[37] Reig M Forner A Rimola J Ferrer-Fabrega J Burrel M Garcia-Criado A . BCLC strategy for prognosis prediction and treatment recommendation: The 2022 update. J Hepatol. (2022) 76:681–93. doi: 10.1016/j.jhep.2021.11.018 34801630 PMC8866082

[38] Singal AG Llovet JM Yarchoan M Mehta N Heimbach JK Dawson LA . AASLD practice guidance on prevention, diagnosis, and treatment of hepatocellular carcinoma. Hepatology. (2023) 78:1922–65. doi: 10.1097/HEP.0000000000000466 37199193 PMC10663390

[39] Ali FS Nguyen MH Hernaez R Huang DQ Wilder J Piscoya A . AGA clinical practice guideline on the prevention and treatment of hepatitis B virus reactivation in at-risk individuals. Gastroenterology. (2025) 168:267–84. doi: 10.1053/j.gastro.2024.11.008 39863345

[40] Chan SL Lamarca A Hsu C Moreno V Chan LL Keenan BP . New targets and new drugs for hepatobiliary cancers. J Hepatol. (2026) 84:213–28. doi: 10.1016/j.jhep.2025.08.018 40865646

[41] Gan B Wu L Zhou S Chen Z Wu F Xu L . Comprehensive analysis of publications concerning combinations of immunotherapy and targeted therapies for hepatocellular carcinoma: A bibliometric study. Front Immunol. (2025) 16:1476146. doi: 10.3389/fimmu.2025.1476146 40013134 PMC11860873

